# A practical update on the epidemiology and risk factors for the emergence and mortality of bloodstream infections from real-world data of 3014 hematological malignancy patients receiving chemotherapy

**DOI:** 10.7150/jca.50802

**Published:** 2021-07-25

**Authors:** Shaozhen Chen, Kangni Lin, Qian Li, Xiaofeng Luo, Min Xiao, Minmin Chen, Haojie Zhu, Yongquan Chen, Xueqiong Wu, Yanling Zeng, Yuxin Zhang, Issa Hajji Ally, Jingjing Xu, Jinhua Ren, Zhizhe Chen, Jianda Hu, Ting Yang

**Affiliations:** 1 Fujian Institute of Hematology, Fujian Provincial Key Laboratory on Hematology, Fujian Medical University Union Hospital, Fuzhou 350001, Fujian, P. R. China; 2 Department of Cancer, Fujian Provincial Cancer Hospital, Fuzhou 350014, Fujian, P. R. China; 3 Department of Hematology, The Second Affiliated Hospital of Xiamen Medical College, Xiamen 361021, Fujian, P. R. China; 4 Department of Hematology, Affiliated Nanping First Hospital of Fujian Medical University, Nanping 353000, Fujian, P.R. China; 5 Department of Hematology, Zhongshan Hospital, Fudan University (Xiamen Branch), Xiamen 361015, Fujian, P.R. China

**Keywords:** Bloodstream infections, hematological malignancy, pathogen, resistance, risk factor

## Abstract

**Background:** Bloodstream infection (BSI) is a common and serious complication after patients with hematologic malignancies (HM) receiving chemotherapy. This study examined real-world data seeking to characterize HM BSI and identify risk factors for BSI emergence and mortality.

**Methods:** We retrospectively analyzed the pathogenic epidemiology, antibiotic resistance, and BSI risk factors in a single-center cohort including 3014 consecutive patients with HM receiving chemotherapy between 2013 and 2016. Results of the pathogenic epidemiology were validated via comparison to available reported data.

**Results:** We found that 725 patients (24.1%) had BSIs. Gram-negative (G-) bacteria represented 64.7% of the 744 isolated pathogenic strains, while Gram-positive (G+) bacteria and fungi accounted for 27.7% and 7.7% of the BSIs, respectively. The most common isolates were *Klebsiella pneumoniae* (19.2%), and 95.1% of the multidrug-resistant strains (MDR) were extended-spectrum beta-lactamase producing strains. G- bacteria were the main microflora responsible for BSI in our cohort of Chinese HM patients compared to studies in developed countries or in neutropenic children with HM or solid tumors. Multivariate analysis revealed that male sex, age ≥ 45 and < 65 yr, hospital length of stay ≥ 9d, neutropenia ≥ 7d before cultures, ≥ 2 antibiotics, and infections (gastrointestinal, perirectal, or urinary tract) independently predicted BSI emergence. Furthermore, age ≥ 65 yr, neutropenia ≥ 7d before blood cultures, no HM remission, lower white blood cell count, ≥ 3 antibiotics, respiratory infections, and *Acinetobacter baumannii* and *Stenotrophomonas maltophilia* BSI were independent predictors of 30-day mortality.

**Conclusions:** G- bacteria were the predominant microflora during the study period and antibiotic resistance levels of the pathogens detected were high, especially for MDR strains. The mortality of BSI patients was high in this large cohort. Close attention should be paid to the risk factors identified here to facilitate timely and effective clinical management of such patients.

## Background

Treatments for hematological malignancies (HM) mainly include radiotherapy, chemo- and targeted-therapy, and hematopoietic stem cell transplantation (HSCT). It is known that chemotherapies and intensive conditioning regimens prior to HSCT can cause mucosal damage, depressed immunity, and neutropenia, all of which can contribute to Bloodstream infection (BSI) development 1-3. Although there have been many great progresses made in recent decades for the treatment of HM, especially the exciting advances in HSCT 4-5, BSIs remain a serious threat to HM patients 3, 6. BSI is a serious complication in patients with HM receiving chemotherapy that result in prolonged hospital length of stay (LOS) 7-8. Furthermore, BSIs induce severe systemic inflammatory reactions, which are associated with high rates of mortality and other poor outcomes 7-10. Thus, the ability to diagnose BSIs early and to treat them in a timely manner could substantially improve patient outcomes.

In this study, we retrospectively collected clinical characteristics data from patients with HM receiving chemotherapy and followed in the Department of Hematology during the period of 1/2013 to 12/2016, and recorded the identified pathogens in such patients who were diagnosed with a BSI. Clinical prognosis was analyzed, and risk factors were determined for this cohort comprising 3014 HM patients and results validated via comparison with available data.

## Materials and Methods

### Setting, patients, and study design

The study involved patients with HMs, followed in the Department of Hematology at the Fujian Medical University Union Hospital, a large general teaching hospital in Fuzhou, China, between January 1, 2013 and December 31, 2016. The medical records of these patients were retrospectively reviewed for the purpose of this study and the local ethics committee ruled that no formal ethics approval was required in this particular cases. Inclusion criteria included: diagnosis with any HM, treatment with chemotherapy, and available blood culture (BC) data. If the patient had two or more positive BC, the clinical data associated with the first positive BC during the same hospitalization was included. A total of 3014 patients fulfilled the inclusion criteria of the study. Patients who did not meet the inclusion criteria were excluded from the study. Data validation was obtained by comparing data from articles published in the past three years on BSI in HM or solid tumor patients, with available data within the manuscript.

Patients were divided into two groups according to the results of the BC, i.e., BSI or non-BSI. The "follow-up" period of the data analysis started at the time of the first BC. The primary outcome was patient death; the secondary outcome was survival or death at day 30 of the follow-up period.

### Patient characteristics and recorded data

Gender, age, underlying diseases, presence of diabetes mellitus, hospital LOS (in days), clinical laboratory values (counts for white blood cells, absolute neutrophil and platelet counts, and hemoglobin levels) at the same time or within 24 hours of BC, neutropenia duration prior to obtaining any BC, disease status (remission or no-remission), number of chemotherapy cycles, strains of pathogenic bacteria and resistance to antibiotics, co-infections (oral, respiratory, gastrointestinal, skin/soft tissue, perirectal, and urinary tract), and antibiotic therapy type(s) were extracted from the charts.

### Bacterial isolates and antimicrobial susceptibility

All of the BCs were processed at the hospital laboratory using the same protocol 11 and an automated BACT/ALERT 3D blood culture system (BioMerieux, Marcy-l'Etoile, France). Blood was cultured for 7 days at 37°C and 5% CO_2_ for both aerobic and anaerobic strains. All isolates were identified by the VITEK2 automated system (BioMerieux, Marcy-l'Etoile, France) and were stored at -80°C for antimicrobial susceptibility testing. Antimicrobial susceptibility to cefoxitin, cefotaxime, cefazolin, cefaclor, cefotiam, cefminox, ceftriaxone, cefpodoxime, ceftazidime, cefepime, gentamicin, high-level gentamicin, high-level streptomycin, cefoperazone/sulbactam, cip-rofloxacin, moxifloxacin, levofloxacin, amoxicillin, ampicillin, piperacillin, penicillin G, oxacillin, amoxicillin/clavulanicacid, piperacillin/tazobactam, ampicillin/sulbactam, trimethoprim-sulfamethoxazole, cefotetan, aztreonam, imipenem, meropenem, amikacin, tobramycin, ciprofloxacin, norfloxacin, rifampicin, quintuptine/dalfoptin, chloramphenicol, tetracycline, tigecycline, clindamycin, erythromycin, linezolid, vancomycin, teicoplanin, and minocycline were evaluated through agar dilution and microdilution methods according to the Clinical and Laboratory Standards Institute (CLSI) guidelines 12. *Escherichia coli* ATCC 25922, *Klebsiella pneumoniae* ATCC 700603, *Pseudomonas aeruginosa* ATCC 27853, *Staphylococcus aureus* ATCC 29213, and *Streptococcus pneumoniae* ATCC 49619 were used for quality control according to the CLSI guidelines.

### Definitions

Hematological malignancies of this study included acute myeloid leukemia, acute lymphoblastic leukemia, chronic myeloid leukemia, chronic lymphocytic leukemia, Hodgkin's lymphoma, non-Hodgkin's lymphoma, multiple myeloma, and myelodysplastic syndromes. Neutropenia was defined as an absolute neutrophil count of ≤ 0.5x10^9^/L, or ≤ 1.0x10^9^/L with a predictable decline of ≤ 0.5x10^9^/L within 24-48 hours 13. MDR was defined as bacteria being resistant to 3 or more classes of antibiotics 14. Extensively drug-resistant was defined as non-susceptible to ≥1 agent in all but ≤ 2 antimicrobial classes 15. Poly-microbial bacteremia was defined as BSI caused by at least two different pathogens 16. There are no widely used criteria for the diagnosis of BSI in patients with HM receiving chemotherapy. We used the definitions proposed by Kameda et al. 17 to define a definite or a probable BSI. Briefly, a "definite BSI" was defined as the isolation from at least one BC of a bacterial or fungal pathogen other than common skin contaminants. For common skin contaminants such as *Diphtheroids*, *Bacillus spp*, *Propionibacterium spp*, *coagulase-negative Staphylococci*, *Viridans Streptococci*, *Aerococcus spp*, and *Micrococcus spp* 18-19, detection in 2 or more separate blood cultures was required for a definite BSI diagnosis. Other BC positive cases were defined as probable BSI.

### Statistical analyses

Statistical analyses were performed using SPSS version 23.0. Continuous variables were expressed as medians and ranges and categorical variables using numbers and percent. For continuous variables, Student's t-tests were used to compare differences between groups if the variables were normally distributed, and Mann-Whitney U tests were used when they were non-normally distributed. The Chi-square test or Fisher's exact test were used for categorical variables. The first blood culture initiation date was subtracted from last follow up date to calculate the observation time, which was expressed in days. Univariate and multivariate logistic regression models were used to assess risk factors for the emergence of BSI, producing odds ratios (OR) and 95.0% confidence intervals (CI). Cox proportional hazards regression was used to assess risk factors with 30-day mortality of HM patients with BSIs, generating hazard ratios (HR) and 95.0% confidence intervals. P-values were 2-sided, and a p value of <0.05 was considered to be statistically significant.

## Results

### Characteristics of patients with hematological malignancies

The characteristics of the patients in the entire cohort are summarized in Table 1. Among the 3014 patients with HM, 725 had BSI while 2289 did not. The median age was similar in the BSI and non-BSI groups (43 vs. 44 yr), and BSIs were more often diagnosed in male patients (60.0%). Of note, the hospital LOS was significantly higher in the BSI group (p<0.001). Acute myeloid leukemia was the most common underlying disease in both groups (48.0% Vs 52.1%), and the percentage of diabetes cases was similar in both groups (10.6% Vs 9.9%). The median number of chemotherapy cycles was significantly higher in the BSI group (p=0.084). All 3014 patients were treated with antibiotics, and both groups experienced various co-infections, among which respiratory infections were the most frequent for both groups (80.8% vs. 81.6%). Notably, the proportion of patients who were given ≥ 2 classes of antibiotics was higher among the BSI group (97.8% vs. 90.5 %), as was the proportion given ≥ 3 classes of antibiotics (83.4% vs. 66.4%).

### Frequency of BSI and distribution of major BSI pathogens

The frequency of BSIs was 24.1% (725/3014), and many different pathogenic strains were identified (n=744), with 17 patients having poly-microbial bacteremia. As shown in Table 2, the most common causative pathogens were Gram-negative bacteria (G-; 64.7%), with *Klebsiella pneumoniae* being the most frequent (19.2%). Some Gram-positive (G+; 27.7%) bacteria were also isolated, and the strain most frequently found in BCs was *Coagulase-negative Staphylococci* (CNS) (14.8%). These data clearly emphasize that a large number of pathogens were responsible for the BSIs of this patient population.

### Risk factors for BSI in HM patients receiving chemotherapy

As shown in Table 3, univariate analyses for the entire 3014 patients revealed that male, age ≥ 45 yr, hospital LOS ≥ 9 d, underlying disease, neutropenia ≥ 7d before BC, treatment with ≥ 2 antibiotics, complications such as other infections (oral, gastrointestinal, perirectal, urinary tract) were all independent risk factors for emergence of BSIs. A subsequent multivariate analysis retained all risk factors as being independently associated with BSI, excepting for the type of malignancy and co-infection of the oral cavity.

### Outcomes and risk factors for 30-day mortality in the BSI group

**Figure 1:**
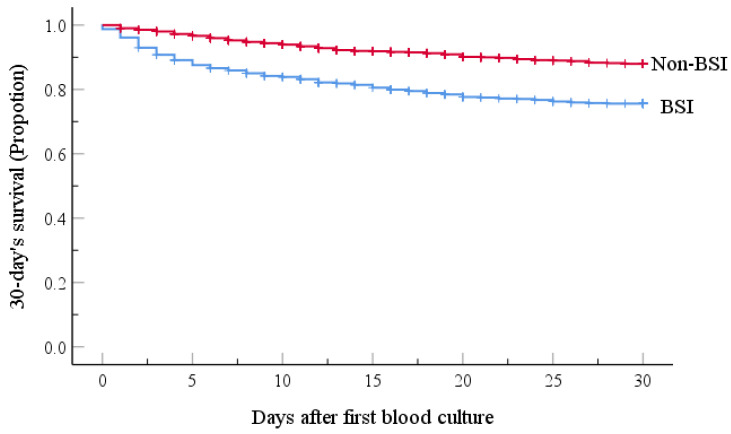
Kaplan-Meier estimates of 30-day patient mortality for the BSI and Non-BSI groups. Kaplan-Meier plots depicting the 30-day survival in patients with hematological malignancy receiving chemotherapy; there was a significant association with the incidence of emergence of BSI. The BSI group had a lower probability of survival (P-value <0.001, by the log-rank method).

The mortality rate at day-30 after BSI onset was 23.6% (171/725) and the BSI group had the lower probability of survival, as shown in Figure 1. Table 4 presents the univariate analyses in the BSI group, which revealed that age ≥ 65 yr, hospital LOS ≥ 9 d, neutropenia ≥ 7 d before BC, lower white blood cell count, lower hemoglobin values, lower platelet counts, no-remission status, respiratory and multi-site co-infections, and treatment with ≥ 3 antibiotic agents were significant risk factors for 30-day mortality. Furthermore, several pathogens responsible for BSIs, including *K. pneumoniae*, *A. baumannii*, *S. maltophilia*, *E. faecium* were also risk factors for 30-day mortality. All risk factors were retained by multivariate analysis as independent predictors of 30-day mortality, except for hospital LOS ≥ 9 d, lower platelet counts, lower hemoglobin values, multi-site co-infections, and *K. pneumoniae* and *E. faecium* BSIs.

### Antimicrobial resistance of major BSI pathogens

**Figure 2:**
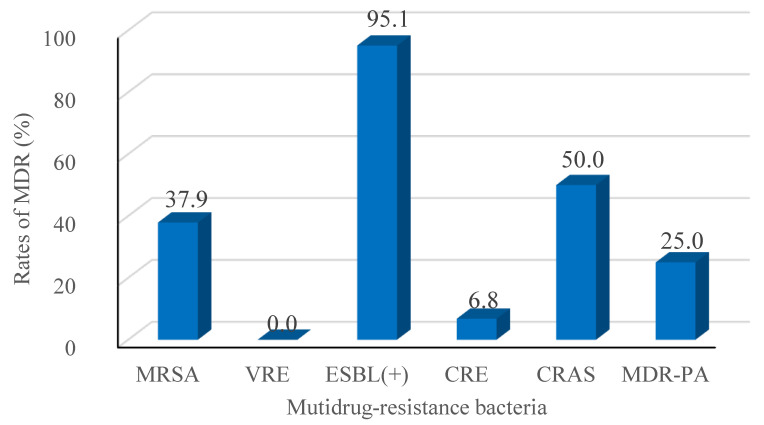
Rates of MDR (%) among the major taxa of pathogens detected from BSIs of the hematological malignancy patients receiving chemotherapy. Following Thaden et al.c, we here defined Multidrug-resistance (MDR) as bacteria being resistant to 3 or more classes of antibiotics as assessed based on antimicrobial susceptibility testing using a Bio Merieux VITEK2 automated system. The tested strains were isolated from blood cultures, and were tested with the more than 40 diverse antibiotics (see Table 5 and 6). MRSA, Methicillin-resistant *Staphylococcus aureus*; VRE, Vancomycin-resistant *Enterococcus*; ESBL (+), extended spectrum β-lactamase-producing; CRE, Carbapenem-resistant *Enterobacterium*; CRAS, Carbapenem-resistant *acinetobacter baumanni*; MDR-PA, multidrug-resistant *Pseudomonas aeruginosa*. c We here defined MDR in reference to Thaden et al.c (Reference: Thaden JT, Li Y, Ruffin F, et al. Increased Costs with Multidrug Resistant Gram Negative Bloodstream Infections Are Primarily Due to Patients with Hospital-Acquired Infections. Antimicrobial Agents & Chemotherapy 2016;61(3):1-10.).

**Figure 3:**
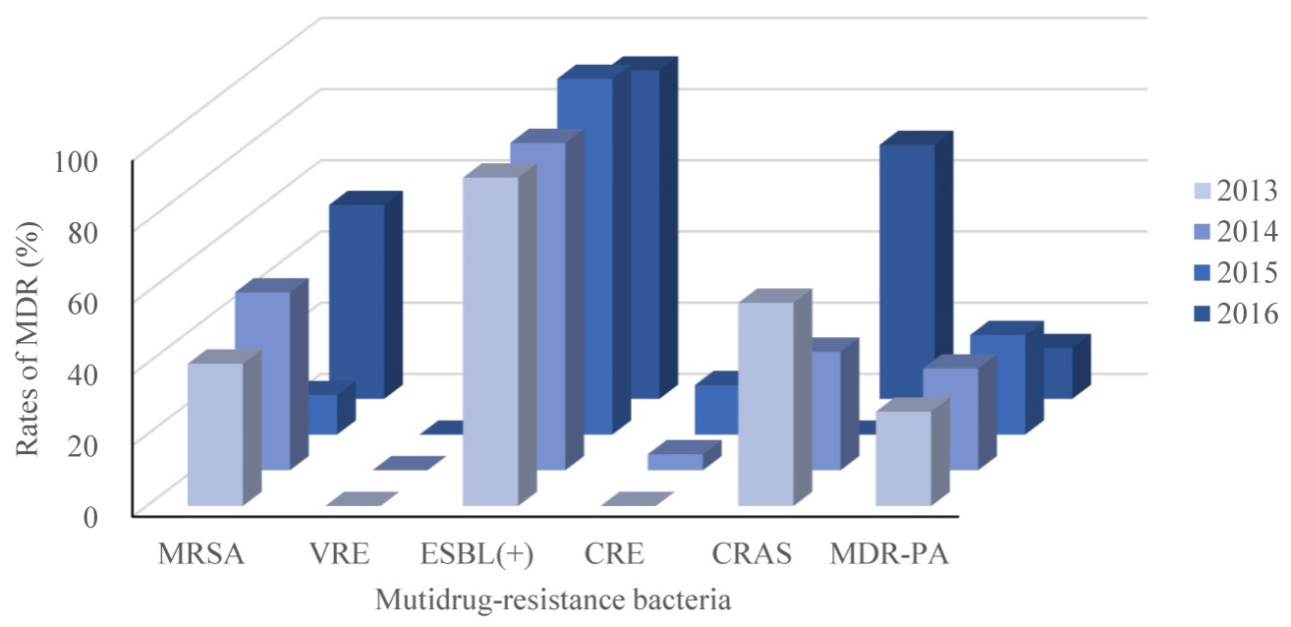
MDR trends between 2013 and 2016. As in Figure 1, but separated by year across the 4 years of this retrospective, single-center study. MRSA, Methicillin-resistant *Staphylococcus aureus*; VRE, Vancomycin-resistant *Enterococcus*; ESBL(+), extended spectrum β lactamase-producing; CRE, Carbapenem-resistant *Enterobacterium*; CRAS, Carbapenem-resistant *Acinetobacter baumanni*; MDR-PA, multidrug-resistant *Pseudomonas aeruginosa*.

Recalling our definition for MDR as resistance to 3 or more classes of antimicrobial agents, our retrospective study identified 327 isolated pathogenic MDR strains out of 744 strains (44.0%). The resistance rates of the main MDR strains were calculated (Figure 2), and the ESBL-producing strains had the highest resistance rate (95.1%; Figure 2). Interestingly, *E. faecium* strains were all classified as MDR, but none were resistant to Vancomycin. No significant differences in the trends for MDR were observed across the four years period of our study, as shown in Figure 3.

Among the *coagulase-negative Staphylococci* strains, 87.2% were resistant to Methicillin; in contrast only 37.9% of the *Staphylococcus aureus* strains were resistant to the Methicillin. The methicillin-resistant *coagulase-negative Staphylococci* strains were generally more likely to exhibit resistance to the tested antibiotics (Table 5), with the exceptions of Gentamycin and Clindamycin, for which methicillin-resistant *Staphylococcus aureus* strains exhibited particularly frequent resistance. Notably, fewer than 50.0% of the *Viridans strains* were resistant to the tested major antibiotics, except for Tetracycline (55.6%) and Erythromycin (60.0%). More than 90.0% of the *E. faecium* strains exhibited resistance to Penicillins, Fluoroquinolones, Erythromycin, and Clindamycin (Table 5). Interestingly, no isolated G+ bacterial strains were resistant to Tigecycline, Linezolid, Vancomycin or Teicoplanin.

The first beta (β) -lactamase was identified in an isolate of *Escherichia coli* in 1940. More than 150 extended-spectrum β-lactamase (ESBL) enzymes types have been identified 20. These enzymes variously confer different types of antibiotic resistance to a range of β-lactamases 21, and they are plasmid-mediated. Evolutionarily, they originate from genetic variants of native β-lactamases found in G- bacteria, especially infectious strains of *Escherichia coli* and *Klebsiella species* 21.The frequencies for ESBL-producing *K. pneumoniae* and *E. coli* strains were 22.6% and 60.0%, respectively. Our antimicrobial susceptibility data from the blood lab indicated that the ESBL-producing *K. pneumoniae* isolates were resistant to most Cephalosporins, although they did not exhibit significant resistance to Cefepime (a fourth generation Cephalosporins class drug). The ESBL-producing *K. pneumoniae* strains were also highly resistant to Ampicillin and to Amoxicillin, which is not sensitive to β-lactamase degradation 22 (Table 6). Resistance to Sulfonamides (64.5%) and to the monobactam class drug Aztreonam (54.8%) was also noted for the ESBL-producing *K. pneumoniae* strains. The non-ESBL-producing *K. pneumoniae* showed some resistance to Cefazolin (77.1%), and were strongly resistant to both Ampicillin and Amoxicillin (100.0%). ESBL-producing *E. coli* strains were resistant to the same antibiotics as the ESBL-producing *K. pneumonia* strains, while the only significant resistance detected for the non-ESBL-producing *E. coli* strains was for Ampicillin (66.7%) and Amoxicillin (60.0%). Fewer than 15.0% of the identified pathogenic *K. pneumoniae* and *E. coli* strains were detected to have resistance to Amikacin, Imipenem, or Tigecycline. *Pseudomonas Aeruginosa* isolates were highly resistant to most Cephalosporins, most semi-synthetic Penicillins, Trimethoprim-sulfamethoxazole (SMZ-TMP), and Tetracycline, but not to Ceftriaxone, Ceftazidime, Cefepime, or Piperacillin. Resistance to other antibiotics was low. *A. Baumannii* was almost totally resistant to most Cephalosporins and to semi-synthetic Penicillins, with resistance to other antibiotics ranging from 40.0-50.0% (Table 6).

### Comparison with the reported data

The epidemiology of BSI in patients with HM has a significant effect on the prognosis. To validate the specific epidemiological characteristics in our center and in the absence of available appropriate databases, we compared our data on the main isolates with those with accessible data in several published studies. Compared to the study by Garcia-Vidal *et al.* in patients with acute leukemia patients 23, our data showed a significant decline of G+ bacteria, especially for CNS (p<0.001), a clear predominance of G- organisms, mainly *K. pneumonia* (p<0.001), and a much higher number of MDR isolates (p<0.001). Furthermore, ESBL-producing *E. coli* tended to be increased (p=0.013), which was not the case for ESBL-producing *K. pneumonia* (p=0.578) (Table 7). Comparing data in neutropenic children with HM, mainly ALL (p<0.001), reported by Zhu Guoqing *et al.* 24 with those in our adult patients (>15 yr), mainly diagnosed with AML, we showed that the dominant microflora was G- bacteria (p<0.001), especially *K. pneumonia* (p<0.001), *P. aeruginosa* (p<0.001), *A. baumannii* (p=0.024) and *S. maltophilia* (p=0.026) (Table 8). Maite *et al.* 25 and Xu Haiyan, *et al.* 26 reported data on solid tumors. Our data indicated that G- bacteria had a higher incidence in BSI with HM than in solid tumors, mainly *E. coli* (p<0.001), *K. pneumonia* (p<0.001) and* P. aeruginosa* (p<0.001) (Table 9).

## Discussion

Infection is the most common complication for patients with HM treated with chemotherapy 19, 27, and is a predictor for adverse outcomes. The most severe infections in these patients are BSIs, probably because of bone marrow suppression, neutropenia 28 and mucosal injury induced by chemotherapy 29. The frequency of BSIs was 24.1% in this study, which is much higher than our previously reported for pre-engraftment patients in Ren et al. (18.3%) 30 or reported for neutropenic patients in Wisplinghoff et al. (14.3%) 7. The higher rate in our study of HM patients receiving chemotherapy may be explained by the fact that all of the HM patients had long-term central venous catheters (CVC) for delivery of chemotherapy. CVCs are well-known risk factors for severe infections, especially BSIs, as bacteria can enter the blood through a CVC 31. Moreover, physicians in our teaching and research hospital have focused their attention on BSIs in recent years, and consequently have more frequently ordered blood cultures from suspected patients, which may have increased the incidence of diagnosed BSI.

Our retrospective, single-center study indicated that G- bacteria were the major BSI pathogens for patients with HM receiving chemotherapy (64.7%), a finding supported by comparing with other public reports 23-26. G-bacteria BSI tended to be the most frequent in Chinese HM patients during the study period, which was similar with the data from the 2013-2016 CHINET surveillance report 32. It indicated that G- bacteria represented above 70.0% of the infection cases in many Chinese general hospitals. Indeed, a previous study reported that G- bacteria were the predominant pathogenic cause of BSI in patients since the 1960s, but that a shift from G- to G+ organisms was observed in the mid-1980s, albeit to a variable extent in different countries. Nevertheless, this trend appears to have been reversed in recent years, with re-emergence of G- bacteria 6, in line with our data. There were significant differences in the BSI isolates, not only between adults and children, but also between solid tumors and HM. The cancer patients accepted chemotherapy or radiotherapy were susceptible to BSI in the combination of mucosal injury, impaired immunity and so on 1, 10. However, there can be cancer-specific in the characteristics of BSI acquisition. In the present study, we found the same result that G- bacteria was more common in patients with HM compared with solid tumors 33.

Plasmid-mediated production of ESBL enzymes by bacteria is a major mechanism of resistance to β-lactamases antibiotics, which include Penicillins, third generation Cephalosporins and single ring β-lactamases antibiotics. There was a high percentage of ESBL-producing strains among the Enterobacteriaceae (22.6% in *K. pneumonia* and 60.0% for *E. coli*). Although not directly comparable, our results appear to be in disagreement with the report by Kara *et al* 34, which included 2098 patients with HM, with 3703 neutropenic episodes over a 5-year period and a rate of ESBL-producing strains of 58.0% for *K. pneumonia* and 45.0% for *E. coli.* Interestingly, we would have expected a greater incidence of G+ bacterial infection, as skin is typically colonized with G+ bacteria 11, 35 and all our patients had long-term CVC for receiving chemotherapy. We also found that compared to other organisms, G- bacteria were present at a significantly greater rate in BSI patients with respiratory (61.3%; 359/586) or gastrointestinal (57.1%; 100/175) co-infection. It bears emphasizing that infections were also observed frequently in the patients of the non-BSI group, indicating that both groups have mucosa damage as a result of their chemotherapy. As the latest Rashidi Armin's report, which demonstrated that specific changes in the gut microbiota precede BSI 36, we speculate this phenomenon may also be associated with G- bacteria colonized in the respiratory and gastrointestinal tracts of HM patients receiving chemotherapy, although this needs to be confirmed by further research.

There have been numerous studies about BSI, but relatively few have reported information about the risk factors for developing BSIs in patients with HM receiving chemotherapy. Some reports investigated patients with catheter-related BSI 37-38, while others reported on patients with BSI after hematopoietic stem cell transplantation 2, 30. It is known that patients with HM undergoing chemotherapy are at high risk of infection, but the specific risk factors for infection were not well defined. Therefore, we compared here the clinical characteristics between BSI and non-BSI patients and found that male, age ≥ 45 yr, hospital LOS ≥ 9 d, neutropenia ≥ 7d before BC, treatment with ≥ 2 antibiotics, complications such as other infections (gastrointestinal, perirectal, urinary tract) were all independent risk factors for emergence of BSI. These results can provide physicians with the means for identifying HM patients at high-risk for BSI who need closer attention and early intervention to curb the occurrence of infection, thereby improving their prognosis.

The 30-day mortality rate in the BSIs group was 23.6%, similar to that reported by Yishu Tang. et al (23.8%) 39. Furthermore, older age, longer hospital LOS, exposure to multiple antibiotics, and neutropenia were not only independent risk factors for emergence of BSIs. In addition, the risk factors describe here can predict the 30-day mortality and have been reported in previous studies 40. Of note, we found that infection with the *A. Baumannii* strains was an independent risk factor for an unfavorable prognosis. This may relate to the high rates of antibiotic resistance among *A. Baumannii* strains—50.0% of which were for example resistant to Carbapenem. Moreover, these findings mirror reports of a significant increasing trend in *A. Baumannii* antibiotic resistance as CHINET detected a rise from 60.0% in 2013 to 70.0% in 2016 32.

The rapid rise of antibiotic resistance makes treatment significantly more difficult and increases mortality 41-42. In our study, both G- and G+ bacteria exhibited extensive resistance to a variety of antibiotics (Table 5 & 6). Although none of the isolates was classified as extensively drug-resistant (XDR), a large number of the ESBL+ strains were MDR (95.1%), which is an alarming high proportion 20-21.

Our detection of Carbapenem-resistant *K. pneumonia* (17.5%) and *E. coli* (4.2%) is particularly worrisome. A systematic review of worldwide reports and meta-analysis demonstrated that the rates of Carbapenem-resistant bacteria in BSIs varied widely across studies, ranging from 2.0% to 33.0% (median 12.0%). Interestingly, the prevalence of Carbapenem resistance among G- bacteria isolated from BSIs in neutropenic patients in China was only 2.0% 43, indicating that Carbapenems remain a relatively effective agent for treating* K. pneumonia* and *E. coli* BSIs in China.

Our study has some limitations. First, it was conducted among inpatients and did not include any outpatients. Thus, some data on possible BSI patients with HM may have been missed. Second, missing values for some variables in the retrospective dataset could have resulted in under- or over-estimation of the results of interest. Third, although unlikely, it is possible that some misclassification bias occurred, due to the rapid death of patients after collecting blood culture samples. This may affect statistics relating to mortality; for example, a patient died 1-2 days after a blood culture sample was sent for examination, but death may have been due to heart issues or recurrence of the primary disease. Nevertheless, these data were not removed and were included in the 30-day mortality rates. Furthermore, these data need to be further confirmed by prospective multicenter studies.

Our results emphasize that timely antibiotic administration for high-risk patients and implementation of evidence-based preventive procedures should be considered when seeking to reduce the development of BSIs and improve outcomes for HM patients receiving chemotherapy. BSIs cause a series of severe systemic inflammatory reactions and progress rapidly, resulting in high mortality rates 8. This study clearly identifies risk factors for developing BSIs and highlights the poor outcomes for HM patients receiving chemotherapy. We also show that all major bacterial pathogens resulting in BSIs were highly resistant to antibiotics, in particular those of the β-lactamases class.

Our large retrospective study using real-world data strongly emphasizes that careful BSI-specific planning and management is needed for HM patients as they undergo chemotherapy and to facilitate close monitoring to help determine their proper course of treatment with suitable antibiotics.

## Figures and Tables

**Table 1:** Characteristics of 3014 HM patients receiving chemotherapy between 2013 and 2016.

Characteristics	BSIs, No. (%) n=725	Non-BSIs, No. (%) n=2289	P value
**Gender**			0.016
Male	435(60.0)	1257(54.9)	
Female	290(40.0)	1032(45.1)	
**Median age, years (range)**	43.0(11.0-86.0)	44.0(7.0-84.0)	<0.001
<45	389(53.7)	1157(50.5)	
≥45, <65	286(39.4)	856(37.4)	
≥65	50(6.9)	276(12.1)	
**Median hospital LOS, days (range)**	23.0(1.0-116.0)	17.0(1.0-94.0)	<0.001
<9	79(10.9)	619(27.0)	
≥9	646(89.1)	1670(73.0)	
**Underlying disease**			<0.001
AML	348(48.0)	1192(52.1)	
ALL	212(29.2)	352(15.4)	
CML	17(2.3)	30(1.3)	
CLL	6(0.8)	33(1.4)	
MDS	20(2.8)	98(4.3)	
MM	13(1.8)	124(5.4)	
NHL	90(12.4)	390(17.0)	
HL	3(0.4)	39(1.7)	
Other	16(2.2)	31(1.4)	
**Duration of neutropenia before BC, days**			<0.001
<7	523(72.1)	1984(86.7)	
≥7	202(27.9)	305(13.3)	
Disease status			0.913
Remission	155(21.4)	485(21.2)	
No remission	570(78.6)	1804(78.8)	
**Median chemotherapy cycles (range)**	3.0(1.0-36.0)	2.0(1.0-43.0	0.084
**Diabetes mellitus**			0.583
Presence	77(10.6)	226(9.9)	
Absence	648(89.4)	2063(90.1)	
**Co-infections**			
**Oral**			0.001
Presence	180(24.8)	435(19.0)	
Absence	545(75.2)	1854(81.0)	
** Respiratory**			0.638
Presence	586(80.8)	1868(81.6)	
Absence	139(19.2)	421(18.4)	
** Gastrointestinal**			<0.001
Presence	175(24.1)	331(14.5)	
Absence	550(75.9)	1958(85.5)	
** Skin and soft tissue**			0.156
Presence	67(9.2)	174(7.6)	
Absence	658(90.8)	2115(92.4)	
** Perirectal**			<0.001
Presence	97(13.4)	183(8.0)	
Absence	628(86.6)	2106(92.0)	
** Urinary tract**			<0.001
Presence	32(4.4)	46(2.0)	
Absence	693(95.6)	2243(98.0)	
** Other locations**			0.499
Presence	20(2.8)	53(2.3)	
Absence	705(97.2)	2236(97.7)	
**Use of antibiotics**			
** ≥2 agents**			<0.001
Done	709(97.8)	2072(90.5)	
Not done	16(2.2)	217(9.5)	
** ≥3 agents**			<0.001
Done	605(83.4)	1521(66.4)	
Not done	120(16.6)	768(33.6)	
**30-day outcome**			<0.001
Survival	554(76.4)	2029(88.6)	
Death	171(23.6)	260(11.4)	

Data are expressed as mean (range) or percent (%). Continuous and categorical variables were compared using Student's t test (or Mann-Whitney U test) and Chi-square test (or Fisher's exact test), respectively. Abbreviations: HM, hematologic malignancies; BSI, bloodstream infection; LOS, length of stay; AML, acute myeloid leukemia; ALL, acute lymphoblastic leukemia; CML, chronic myeloid leukemia; CLL, chronic lymphocytic leukemia; MDS, myelodysplastic syndromes; MM, multiple myeloma; NHL, non-Hodgkin's lymphoma; HL, Hodgkin's lymphoma; BC, blood culture. All inferential statistical test information goes here.

**Table 2:** Distribution of pathogenic strains in blood samples from the BSI group.

Pathogens	Isolates, No. (%) n=744
**Gram-negative bacteria**	**481(64.7)**
*K. pneumonia*	143(19.2)
* E. coli*	122(16.4)
* P. aeruginosa*	108(14.5)
* E. cloacae*	23(3.1)
* A. baumannii*	22(3.0)
* S. maltophilia*	16(2.2)
Other	47(6.3)
**Gram-positive bacteria**	**206(27.7)**
CNS	110(14.8)
*S. aureus*	29(3.9)
* Viridans S.*	35(4.7)
* E. faecium*	15(2.0)
Other	17(2.3)
**Fungi**	**57(7.7)**

Abbreviations: BSI, bloodstream infection; *K. pneumonia, Klebsiella pneumonia; E. coli, Escherichia coli; P. aeruginosa, Pseudomonas aeruginosa; E. cloacae, Enterobacter cloacae; A. baumannii, Acinetobacter baumannii; S. maltophilia, Stenotrophomonas maltophilia; CNS, Coagulase-negative Staphylococci; S. aureus, Staphylococcus aureus; Viridans S., Viridans Streptococci; E. faecium, Enterococcus faecium*.

**Table 3:** Univariate and multivariate analyses of risk factors for emergence of BSIs.

Factor	BSIs	Non-BSIs	Univariate analyses		Multivariate analyses	
	(n=725)	(n=2289)	OR(95%CI)	P-value	OR(95%CI)	P-value
**Gender**						
Female	290	1032	1(ref)		1(ref)	
Male	435	1257	1.232(1.039-1.460)	0.016	1.321(1.102-1.585)	0.003^a^
**Age, years**						
<45	389	1157	1(ref)		1(ref)	
≥45, <65	286	856	0.994(0.833-1.185)	0.944	1.232(1.011-1.501)	0.038^a^
≥65	50	276	0.539(0.390-0.744)	<0.001	0.716(0.505-1.015)	0.060
**Hospital LOS, days**						
<9	79	619	1(ref)		1(ref)	
≥9	646	1670	3.031(2.358-3.896)	<0.001	2.050(1.556-2.702)	<0.001^a^
**Underlying disease**						
AML	348	1192	0.566(0.306-1.046)	0.069	0.522(0.274-0.993)	0.048
ALL	212	352	1.167(0.623-2.184)	0.629	1.101(0.570-2.128)	0.775
CML	17	30	1.098(0.471-2.562)	0.829	0.990(0.407-2.406)	0.982
CLL	6	33	0.352(0.122-1.015)	0.053	0.469(0.157-1.405)	0.176
MDS	20	98	0.395(0.183-0.855)	0.018	0.333(0.149-0.744)	0.007
MM	13	124	0.203(0.088-0.466)	<0.001	0.217(0.092-0.512)	0.001
NHL	90	390	0.447(0.234-0.853)	0.015	0.505(0.257-0.990)	0.047
HL	3	39	0.149(0.040-0.558)	0.005	0.274(0.071-1.060)	0.061
Other	16	31	1(ref)		1(ref)	
**Duration of neutropenia before BC, days**						
<7	523	1984	1(ref)		1(ref)	
≥7	202	305	2.512(2.052-3.075)	<0.001	1.756(1.410-2.186)	<0.001^a^
**Use of antibiotics**						
**≥2 agents**						
Not done	16	217	1(ref)		1(ref)	
Done	709	2072	4.641(2.773-7.766)	<0.001	2.428(1.386-4.255)	0.002^a^
**≥3 agents**						
Not done	120	768	1(ref)		1(ref)	
Done	605	1521	2.546(2.055-3.154)	<0.001	1.393(1.080-1.797)	0.011^a^
**Co-infections**						
**Oral**						
Absence	545	1854	1(ref)		1(ref)	
Presence	180	435	1.408(1.155-1.716)	<0.001	1.053(0.851-1.304)	0.633
** Gastro-intestinal**						
Absence	550	1958	1(ref)		1(ref)	
Presence	175	331	1.882(1.532-2.313)	<0.001	1.450(1.163-1.807)	0.001^a^
** Perirectal**						
Absence	628	2106	1(ref)		1(ref)	
Presence	97	183	1.778(1.368-2.309)	<0.001	1.539(1.164-2.035)	0.002^a^
** Urinary tract**						
Absence	693	2243	1(ref)		1(ref)	
Presence	32	46	2.252(1.423-3.564)	<0.001	2.073(1.268-3.389)	0.004^a^

Abbreviations: BSI, bloodstream infection; OR, odds ratio; CI, confidence interval; LOS, length of stay; AML, acute myeloid leukemia; ALL, acute lymphoblastic leukemia; CML, chronic myeloid leukemia; CLL, chronic lymphocytic leukemia; MDS, myelodysplastic syndromes; MM, multiple myeloma; NHL, non-Hodgkin's lymphoma; HL, Hodgkin's lymphoma; BC, blood culture. ^a^ Statistically significant

**Table 4:** Univariate and multivariate analyses of risk factors associated with 30-day mortality among patients with BSIs.

Factor	Survived	Died	Univariate analyses		Multivariate analyses	
	(n=554)	(n=171)	HR(95%CI)	P-value	HR(95%CI)	P-value
**Age, years**						
<45	310	79	1(ref)		1(ref)	
≥45, <65	213	73	1.317(0.958-1.810)	0.090	1.393(0.997-1.945)	0.052
≥65	31	19	2.075(1.257-3.424)	0.004	1.800(1.070-3.028)	0.027^a^
**Hospital LOS, days**						
<9	54	25	1(ref)		1(ref)	
≥9	500	146	0.600(0.392-0.917)	0.018	0.212(0.128-0.350)	<0.001
**Duration of neutropenia before BC, days**						
<7	425	98	1(ref)		1(ref)	
≥7	129	73	2.192(1.618-2.969)	<0.001	1.449(1.016-2.066)	0.040^a^
**Hemograms of BSIs**						
WBC (10^9^/L), median (range)	0.3(0.01-157.80)	0.25(0.01-918.39)	1.004(1.002-1.006)	<0.001	1.003(1.001-1.005)	0.012^a^
Hb (g/L), median (range)	65(26-142)	56(29-120)	0.976(0.967-0.985)	<0.001	0.984(0.974-0.995)	0.003
PLT (10^9^/L), median (range)	17(1-1172)	15(1-213)	0.993(0.988-0.998)	0.008	0.998(0.992-1.003)	0.404
**Disease status**						
Remission	141	14	1(ref)		1(ref)	
No-remission	413	157	3.353(1.941-5.794)	<0.001	2.715(1.482-4.972)	0.001^a^
**Use of antibiotics (≥3 agents)**						
Not done	108	12	1(ref)		1(ref)	
Done	446	159	2.839(1.579-5.105)	<0.001	2.178(1.170-4.056)	0.014^a^
**Co-infections (respiratory)**						
Not done	133	6	1(ref)		1(ref)	
Done	421	165	7.382(3.269-16.672)	<0.001	4.661(1.990-10.917)	<0.001^a^
**Numbers of co-infected locations, median (range)**	1(0-5)	2(0-5)	1.251(1.075-1.455)	0.004	1.021(0.851-1.226)	0.821
**Pathogens for BSIs**						
* K. pneumonia*	89	45	2.065(1.041-4.098)	0.038	1.672(0.783-3.573)	0.184
* P. aeruginosa*	82	24	1.264(0.605-2.644)	0.533	1.056(0.471-2.368)	0.894
* E. coli*	96	18	0.869(0.401-1.882)	0.721	0.778(0.334-1.814)	0.561
CNS	92	15	0.733(0.329-1.631)	0.446	0.590(0.166-2.103)	0.416
* S. sureus*	27	2	0.367(0.080-1.673)	0.195	0.302(0.049-1.860)	0.197
* Viridans S.*	29	3	0.493(0.136-1.790)	0.282	0.429(0.084-2.195)	0.310
* A. baumannii*	10	10	4.082(1.698-9.812)	0.002	4.621(1.744-12.245)	0.002^a^
* S. maltophilia*	6	9	5.100(2.070-12.562)	<0.001	3.724(1.408-9.848)	0.008^a^
* E. faecium*	6	8	4.053(1.599-10.274)	0.003	1.853(0.470-7.305)	0.378
* E. cloacae*	14	7	1.792(0.682-4.708)	0.237	1.192(0.427-3.326)	0.737
* A. hydrophila*	4	1	0.986(0.126-7.700)	0.989	2.124(0.258-17.491)	0.484
Fungi	44	12	1.209(0.522-2.799)	0.657	1.263(0.224-7.128)	0.791
Polymicrobia	10	7	2.790(1.062-7.332)	0.037	3.260(0.986-10.782)	0.053
Other	45	10	1(ref)		1(ref)	
**Organisms**						
Gram-negative	338	125	0.342(0.105-1.120)	0.076	2.198(0.341-14.170)	0.408
Gram-positive	167	31	0.644(0.205-2.024)	0.451	1.850(0.399-8.572)	0.432
Fungi	44	12	0.492(0.139-1.745)	0.272	1.263(0.224-7.128)	0.791
Polyorganisms	5	3	1(ref)		1(ref)	

Abbreviations: BSI, bloodstream infection; HR, hazard ratio; CI, confidence interval; LOS, length of stay; BC, blood culture; WBC, white blood cell; Hb, hemoglobin; PLT, platelets; *K. pneumonia, Klebsiella pneumonia; P. aeruginosa, Pseudomonas aeruginosa; E. coli, Escherichia coli; CNS, Coagulase-negative Staphylococci; S. aureus, Staphylococcus aureus; Viridans S., Viridans Streptococci; A. baumannii, Acinetobacter baumannii; S. maltophilia, Stenotrophomonas maltophilia; E. faecium, Enterococcus faecium; E. cloacae, Enterobacter cloacae; A. hydrophila, aeromonas hydrophila.* ^a^ Statistically significant

**Table 5:** Rates of antibacterial resistance (%) among major taxa for Gram-positive strains isolated from BSI patients.

Antibiotics	CNS (n= 109)^b^		*S. aureus* (n= 29)		*Viridans S.* (n= 35)	*E. faecium* (n= 15)
	MRCNS	MSCNS	MRSA	MSSA		
	87.2	12.8	37.9	62.1		
**Cephalosporins**						
Ceftriaxone	-	-	-	-	11.8	-
Cefotaxime	-	-	-	-	14.3	-
Cefepime	-	-	-	-	9.1	-
**Penicillins**						
Ampicillin	-	-	-	-	9.1	92.9
Penicillin G	100.0	78.6	100.0	94.1	15.8	92.9
Oxacillin	100.0	0	100.0	0	-	-
**Aminoglycosides**						
Gentamicin	15.8	0	27.3	5.6	-	-
High-level gentamicin	-	-	-	-	-	78.6
High-level streptomycin	-	-	-	-	-	50.0
**Fluoroquinolones**						
Ciprofloxacin	58.5	7.7	27.3	5.6	-	100.0
Moxifloxacin	17.9	0	9.1	0	-	92.9
Levofloxacin	38.9	0	18.2	5.6	18.2	92.9
**Sulfonamides**						
Trimethoprim-sulfamethoxazole	63.8	30.8	9.1	11.1	33.3	-
**Tetracyclines**						
Tetracycline	54.7	30.8	36.4	27.8	55.6	57.1
Tigecycline	0	0	0	0	-	0
**Glycopeptides**						
Linezolid	0	0	0	0	0	0
Teicoplanin	0	0	0	0	-	0
Vancomycin	0	0	0	0	0	0
**Macrolides**						
Erythromycin	86.3	42.9	81.8	22.2	60.0	92.9
**Lincosamides**						
Clindamycin	67.0	28.6	72.7	16.7	48.6	100.0
**Others**						
Rifampicin	8.4	7.7	9.1	0	-	-
Quintuptine/dalfoptin	-	-	-	-	-	0
Chloramphenicol	-	-	-	-	16.7	-

Abbreviations: BSI, bloodstream infection; CNS, *Coagulase-negative Staphylococci*; *S. aureus*, *Staphylococcus aureus*; *Viridans S.*, *Viridans Streptococci*; E. faecium, *Enterococcus faecium*; MRCNS, methicillin-resistant *Coagulase-negative Staphylococci*; MSCNS, methicillin-sensitive *Coagulase-negative Staphylococci*; MRSA, methicillin-resistant *Staphylococcus aureus*; MSSA, methicillin-sensitive *Staphylococcus aureus*. ^b^ Strains with available drug resistance data were included in this analysis.

**Table 6:** Rates of antibacterial resistance (%) among major taxa for Gram-negative strains isolated from BSI patients.

Antibiotics	*K. pneumonia* (n= 140)^b^		*E. coli* (n= 120)^b^		*P. aeruginosa* (n= 107)^b^	*A. baumannii* (n= 21)^b^	*S. maltophilia* (n= 14)^b^
	ESBL(+)	ESBL(-)	ESBL(+)	ESBL(-)			
	22.6	77.4	60.0	40.0			
**Cephalosporins**							
Cefazolin	100.0	77.1	100.0	40.0	100.0	100.0	-
Cefaclor	100.0	47.5	100.0	16.7	100.0	100.0	-
Cefoxitin	16.1	25.0	16.9	7.3	100.0	100.0	-
Cefotiam	-	-	-	-	100.0	-	-
Cefminox	100.0	4.7	100.0	2.2	100.0	100.0	-
Cefpodoxime	100.0	20.2	100.0	2.3	100.0	100.0	-
Cefatriaxone	90.3	19.3	98.6	4.2	0	50.0	-
Moxalactam	-	0	-	0	-	-	-
Cefotaxime	88.9	20.2	98.5	4.3	-	100.0	-
Ceftazidime	100.0	19.2	30.0	4.2	5.0	90.0	-
Cefotetan	-	-	-	-	100.0	100.0	-
Cefepime	19.3	17.4	30.6	4.2	6.5	50.0	-
**Semi-synthetic penicillins**							
Amoxicillin	100.0	100.0	100.0	60.0	100.0	100.0	-
Ampicillin	100.0	100.0	100.0	66.7	100.0	100.0	-
Piperacillin	-	-	-	-	5.3	-	-
**Aminoglycosides**							
Amikacin	6.4	8.3	8.3	0	0.9	-	-
Tobramycin	6.5	12.8	29.2	4.2	0.9	40.0	-
Gentamicin	54.8	15.6	59.2	25.0	1.9	50.0	-
**Fluoroquinolones**							
Ciprofloxacin	29.0	18.3	70.8	22.9	2.8	45.0	-
Norfloxacin	-	-	-	-	2.1	0	-
Levofloxacin	25.8	15.6	66.7	22.9	2.8	35.0	14.3
**Sulfonamides**							
Trimethoprim-sulfamethoxazole	64.5	18.5	76.4	54.2	100.0	45.0	0
**Carbapenems**							
Imipenem	3.2	14.3	0	4.2	14.0	50.0	-
Meropenem	-	-	-	-	11.6	-	-
**Tetracycline**							
Tigecycline	10.0	3.1	0	0	100.0	0	-
Minocycline	-	-	-	-	100.0	42.9	0
**Beta-lactamases**							
Aztreonam	54.8	15.6	59.7	2.1	-	100.0	-
**Others**							
Cefoperazone/sulbactam	-	-	-	-	0	71.4	-
Amoxicillin/clavulanic acid	16.1	27.3	12.3	9.5	88.9	100.0	-
Paracillin/tazobactam	6.5	19.4	2.8	4.2	3.7	50.0	-
Ampicillin/sulbactam	-	-	-	-	100.0	50.0	-

Abbreviations: BSI, bloodstream infection; *K. pneumonia*, *Klebsiella pneumonia*; *E. coli*, *Escherichia coli*; *P. aeruginosa*, *Pseudomonas aeruginosa*; *A. baumannii*, *Acinetobacter baumannii*; *S. maltophilia*, *Stenotrophomonas maltophilia*, ESBL(+), extended-spectrum β-lactamase producing; ESBL(-), non-extended-spectrum β-lactamase producing. ^b^ Strains with available drug resistance data were included in this analysis.

**Table 7:** G- bacteria were the dominant microflora of BSI with acute leukemia in the Chinese population compared to Spain.

	Reported study (Garcia-Vidal* et al*.) 23				Our study	
	2004-2007 n=233(%)	2008-2011 n=215(%)	2012-2016 n=141(%)	P-value (Raw data)	2013-2016 n=575(%)	P-value (Compared data)
**Gender, male**	324(55.0)				331(57.6)	0.408
**Median age, years (IQR)**	53.0(40.5-64)				39.5(27-52)	NA
**Main isolates**						
**G- bacteria**	92(39.5)	103(47.9)	60(42.6)	0.405	380(66.1)	<0.001^a^
*K. pneumonia*	8(3.4)	13(6.0)	7(5.0)	0.404	114(19.8)	<0.001^a^
ESBL(+)	6(2.6)	3(1.4)	1(0.7)	0.161	17(3.0)	0.578
* E. coli*	54(23.2)	39(18.1)	27(19.1)	0.280	108(18.8)	0.208
ESBL(+)	15(6.4)	8(3.7)	8(5.7)	0.611	61(10.6)	0.013^a^
* P. aeruginosa*	19(8.2)	35(16.3)	17(12.1)	0.141	79(13.7)	0.080
MDR	10(4.3)	20(9.3)	11(7.8)	0.125	18(3.1)	0.149
**G+ bacteria**	137(58.8)	113(52.6)	73(51.8)	0.151	149(25.9)	<0.001^a^
CNS	97(41.6)	74(34.4)	40(28.4)	0.008	82(14.3)	<0.001^a^
* S. aureus*	10(4.3)	6(2.8)	3(2.1)	0.229	18(3.1)	0.476
MRSA	2(0.9)	2(0.9)	0	0.378	7(1.2)	0.627
**MDR isolates**	43(18.5)	44(20.5)	23(16.3)	0.703	257(44.7)	<0.001^a^

Abbreviations: BSI, bloodstream infection; IQR: Interquartile range; NA, Not applicable; G-, Gram-negative; *K. pneumonia*, *Klebsiella pneumonia*; ESBL(+), extended-spectrum β-lactamase producing; *E. coli*, *Escherichia coli*; *P. aeruginosa*, *Pseudomonas aeruginosa*; MDR, multidrug-resistance; G+, Gram-positive; CNS, Coagulase-negative *Staphylococci*; *S. aureus*, *Staphylococcus aureus*; MRSA, methicillin-resistant *Staphylococcus aureus*. ^a^ Statistically significant

**Table 8:** G- bacteria were the dominant microflora of BSI in neutropenic adult HM compared with neutropenic children (≤15 years) in Chinese population.

	Our study (n=574)	Reported study (Zhu Guoqing* et al*.)^24^ (n=427)	P-value
**Median age, years**	42	8	
**Gender, male**	348	254	0.744
**Underlying disease**			<0.001^a^
ALL	184	241	
AML	280	168	
MDS	18	5	
Lymphoma	61	4	
Other	31	9	
**Main isolates**			
**G- bacteria**	394	253	<0.001^a^
* K. pneumonia*	126	67	<0.001^a^
* E. coli*	104	99	0.869
* P. aeruginosa*	86	28	<0.001^a^
* E. cloacae*	19	9	0.084
* A. baumannii*	16	5	0.024^a^
*S. maltophilia*	14	4	0.026^a^
**G+ bacteria**	139	281	<0.001
* S. aureus*	21	32	0.070
* S. epidermidis*	16	75	<0.001^a^
* Viridans S.*	28	109	<0.001^a^

Abbreviations: BSI, bloodstream infection; HM, hematologic malignancies; ALL, acute lymphoblastic leukemia; AML, acute myeloid leukemia; MDS, myelodysplastic syndromes; G-, Gram-negative; *K. pneumonia, Klebsiella pneumonia; E. coli, Escherichia coli; P. aeruginosa, Pseudomonas aeruginosa; E. cloacae, Enterobacter cloacae; A. baumannii, Acinetobacter baumannii; S. maltophilia, Stenotrophomonas maltophilia;* G+, Gram-positive;* S. aureus, Staphylococcus aureus; S. epidermidis, Staphylococcus epidermidis; Viridans S., Viridans Streptococci.* ^a^ Statistically significant

**Table 9:** G- bacteria were the dominant microflora of BSI with HM compared with foreign and domestic solid tumor patients.

	Our study n=725	Foreign reported study (Maite. *et al*)^25^ n=742	Domestic reported study (Xu Haiyan. *et al*)^26^ n=220	P-value
**Median ages, years (range)**	43.0(11-86)	62.7(14-85)	58.6	NA
**Gender, male**	435	460	137	0.454
**Main isolates**				
**G- bacteria**	481	428	102	<0.001^a^
* K. pneumonia*	143	77	24	<0.001^a^
* E. coli*	122	240	50	<0.001^a^
* P. aeruginosa*	108	68	6	<0.001^a^
**G+ bacteria**	206	233	100	<0.001^a^
CNS	110	31	60	0.299
*S. aureus*	29	53	17	0.010^a^
MRSA	11	9	NA	0.657
*Viridans S.*	35	43	NA	0.089
** MDR isolates**	324	94	NA	<0.001^a^

Abbreviations: BSI, bloodstream infection; HM, hematologic malignancies; NA, Not applicable; G-, Gram-negative; *K. pneumonia*, *Klebsiella pneumonia*; *E. coli*, *Escherichia coli*; *P. aeruginosa*, *Pseudomonas aeruginosa*; G+, Gram-positive; CNS, Coagulase-negative Staphylococci; *S. aureus*, *Staphylococcus aureus*; MRSA, methicillin-resistant *Staphylococcus aureus*; *Viridans S*., *Viridans Streptococci*; MDR, multidrug-resistance. ^a^ Statistically significant
